# Emergence of Visual Saliency from Natural Scenes via Context-Mediated Probability Distributions Coding

**DOI:** 10.1371/journal.pone.0015796

**Published:** 2010-12-29

**Authors:** Jinhua Xu, Zhiyong Yang, Joe Z. Tsien

**Affiliations:** 1 Brain and Behavior Discovery Institute, Georgia Health Sciences University, Augusta, Georgia, United States of America; 2 Department of Computer Science and Technology, East China Normal University, Shanghai, China; 3 Department of Ophthalmology, Georgia Health Sciences University, Augusta, Georgia, United States of America; 4 Department of Neurology, Georgia Health Sciences University, Augusta, Georgia, United States of America; University of Maribor, Slovenia

## Abstract

Visual saliency is the perceptual quality that makes some items in visual scenes stand out from their immediate contexts. Visual saliency plays important roles in natural vision in that saliency can direct eye movements, deploy attention, and facilitate tasks like object detection and scene understanding. A central unsolved issue is: What features should be encoded in the early visual cortex for detecting salient features in natural scenes? To explore this important issue, we propose a hypothesis that visual saliency is based on efficient encoding of the probability distributions (PDs) of visual variables in specific contexts in natural scenes, referred to as context-mediated PDs in natural scenes. In this concept, computational units in the model of the early visual system do not act as feature detectors but rather as estimators of the context-mediated PDs of a full range of visual variables in natural scenes, which directly give rise to a measure of visual saliency of any input stimulus. To test this hypothesis, we developed a model of the context-mediated PDs in natural scenes using a modified algorithm for independent component analysis (ICA) and derived a measure of visual saliency based on these PDs estimated from a set of natural scenes. We demonstrated that visual saliency based on the context-mediated PDs in natural scenes effectively predicts human gaze in free-viewing of both static and dynamic natural scenes. This study suggests that the computation based on the context-mediated PDs of visual variables in natural scenes may underlie the neural mechanism in the early visual cortex for detecting salient features in natural scenes.

## Introduction

Detecting salient features and objects in complex natural scenes is indispensible to any visual system. Visual saliency plays important roles in natural vision in that saliency can direct eye movement, deploy attention, facilitate tasks like object detection and scene understanding, and help determine internal neural representation. Not surprisingly, human vision has an amazing ability to detect salient objects in complex natural scenes in real time despite the limited resources of the human visual system.

Visual saliency is closely related to several areas of vision research performed during the last 30 years, including: non-classical receptive fields and contextual effects on neuronal responses [1], [2], texture perception (e.g., the texton theory [3]), pop-out and visual search (e.g., the feature integration theory [4] and the guided search theory [5]), saliency-based attention [6], and neuronal responses to natural scenes [7]. At the center of these areas of research are two issues: what visual features should be encoded in the visual cortex and how they give rise to visual saliency. The conventional view that neurons in the early visual cortex encode individual visual features cannot account for a range of observations in these research areas. This quandary has led to a burgeoning interest in the statistics of natural environments and their relationship to vision [8], [9]. The underlying assumption is that the visual system must inevitably adapt, by evolution and individual development, to the statistical characteristics of the environments that their possessors inhabit [10], [11]. In particular, the efficient coding hypothesis holds that the purpose of early visual processing is to generate efficient representations of visual stimuli [12]–[14]. Similarly, the receptive fields of simple and complex cells can be derived based on this hypothesis [15]–[20] and the responses of V1 neurons in awake, behaving macaques suggest that classical and non-classical RFs form a sparse representation of the visual world [21]. Despite these efforts, it remains unclear what visual features in natural visual scenes should be encoded and how they give rise to visual saliency [2], [22].

Several computational models of visual saliency have been developed [23]–[35]. In Itti *et al*'^s^ model [23], [24], a measure of saliency is computed based on the relative difference between a target and its surround along a set of feature dimensions (i.e., color, intensity, orientation, and motion) obtained by filtering. Zhaoping developed a neural dynamic model in which visual saliency is computed as an index of local neuronal population responses [25], [26], suggesting that a separate saliency map in the brain suggested by Koch & Ullman [27] may not be necessary. Several statistical models of visual saliency have also been developed [30]–[35]. In these models, a set of statistics or PDs are computed from either the scene the subject is viewing or a set of natural scenes, and a variety of measures of visual saliency are defined on these statistics or PDs, including self-information [30], [31], discriminant power [32], [33], Bayesian surprise [34], and inverse of likelihood [35]. These models predict aspects of human gaze in free-viewing natural scenes. However, none of these models provides probabilistic descriptions of a full range of visual variables in natural scenes, so they shed little light on what and how visual variables in natural scenes should be encoded in the early visual cortex.

**Figure 1:**
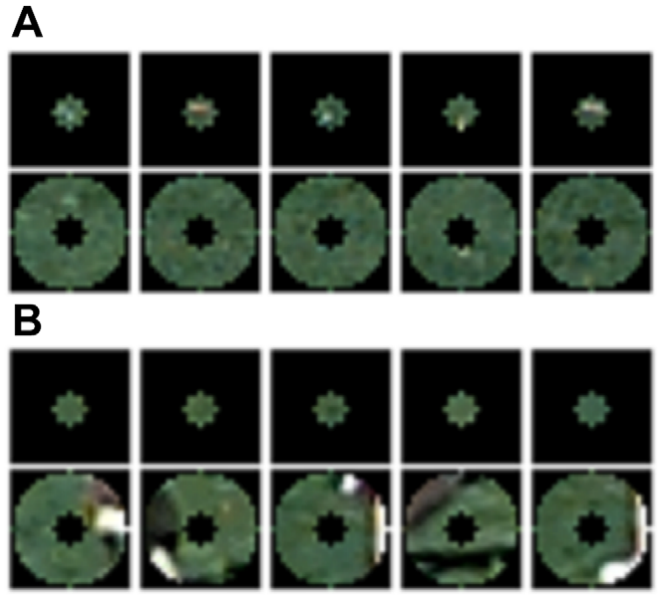
Variations of visual features and co-occurring contexts in natural scenes. (**A**) Similar targets occur in a variety of contexts. (**B**) Various targets occur in similar contexts.

Here, we took a different approach. Since natural visual scenes entail a variety of structured statistics, occurring over the full range of natural variations in the world, a given visual feature could appear in many different ways and in a variety of contexts in natural scenes (Fig. 1). It is conceivable that dealing efficiently with these variations is vital for performing natural tasks. In fact, for visual saliency to have any biological utility for natural vision, it must be tied to the statistics of natural variations of visual features and their contexts. Therefore, we proposed to test a novel hypothesis that visual saliency is based on efficient encoding of the probability of observing visual variables with respect to specific scene contexts. In other words, saliency should be *high* when a visual variable appears with an *unlikely* context; but saliency should be *low* when a visual variable appears with a *likely* context.

To test this hypothesis, we developed a model of context-mediated PDs in natural scenes. In this model, we used a set of conditional PDs based on the independent components (ICs) of natural scenes in a target-context configuration (described later). This target-context configuration was studied in both spatial and temporal domains. We then estimated these PDs from a set of natural scenes and derived a measure of visual saliency. Finally, we conducted an extensive evaluation of this model of visual saliency and found that it is a good predictor of human gaze during the free-viewing of both static and dynamic natural scenes.

## Results

### Context-mediated PDs in natural scenes and visual saliency

**Figure 2:**
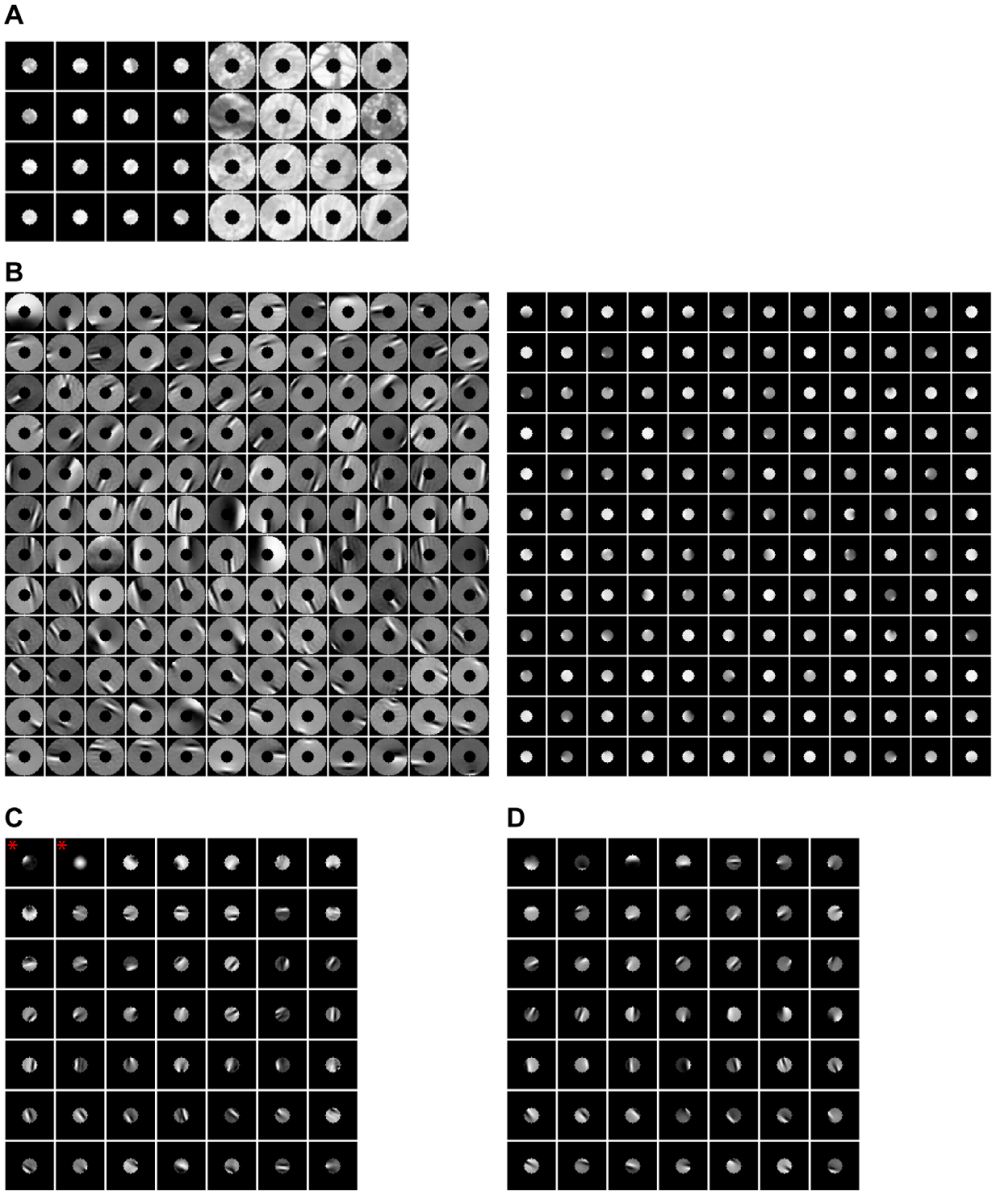
Patches of luminance images of natural scenes and ICs. (**A**) Examples of image patches in a center-surround configuration. (**B**) Examples of paired center and surround ICs. (**C**) Examples of unpaired center ICs. (**D**) Examples of the ICs for the center computed alone.

**Figure 3:**
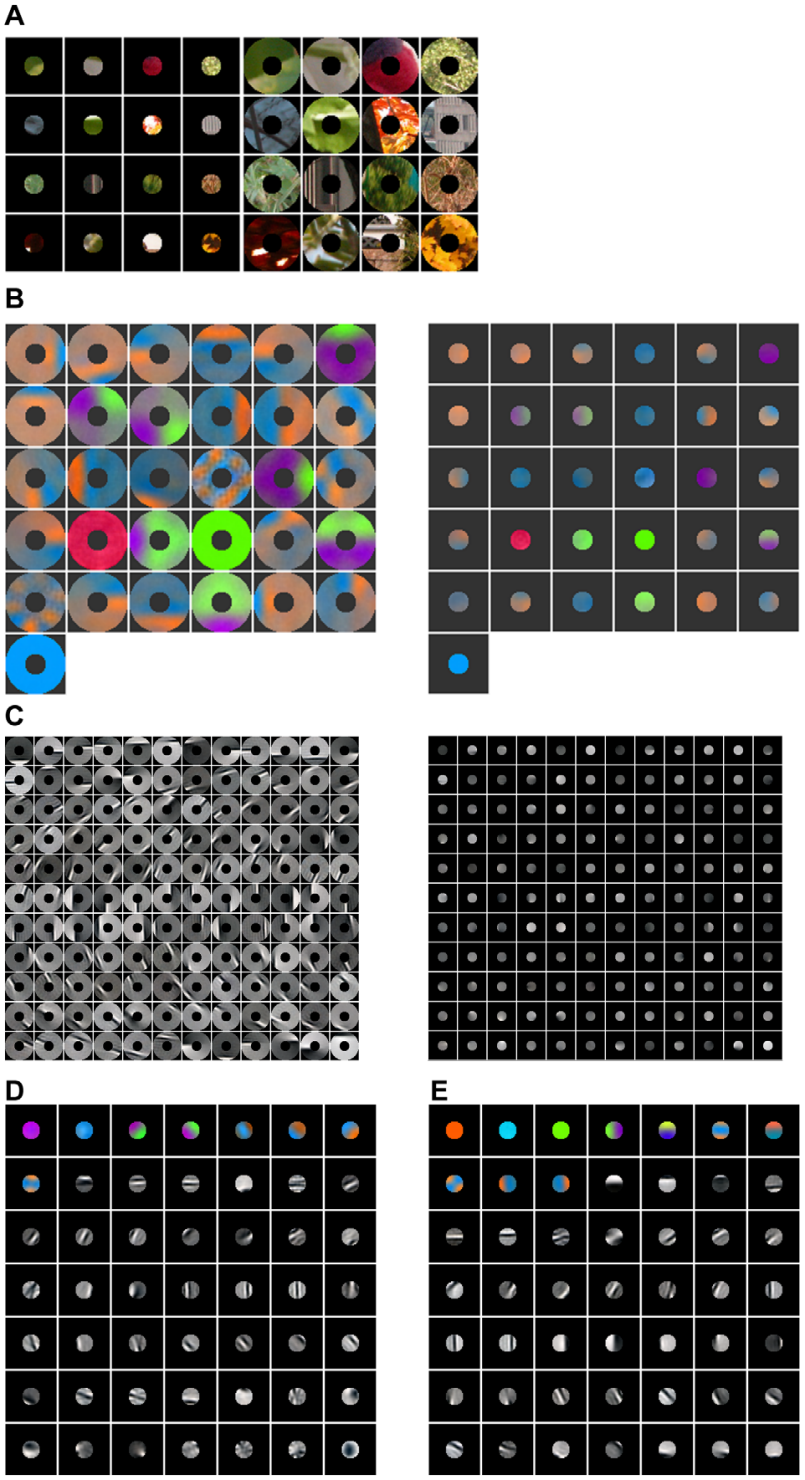
Patches of color images of natural scenes and ICs. (**A**) Examples of color image patches in a center-surround configuration. (**B**) Examples of paired chromatic center and surround ICs. (**C**) Examples of paired achromatic center and surround ICs. (**D**) Examples of unpaired center ICs. (**E**) Examples of the ICs for the center computed alone.



The context-mediated PDs in natural scenes are the conditional PDs of a target for a given context in natural scenes. Here, a context refers to the natural scene patch that co-occurs with a visual target in question in a space and/or time domain. We propose that the context-mediated PDs in natural scenes are represented by ICs of natural scenes. There are several reasons for this hypothesis. First, it has been argued extensively that the early visual cortex represents incoming stimuli in an efficient manner [14]. The distributions of the amplitudes of ICs of natural scenes are highly non-Gaussian with high peaks at zero and long tails, meaning that only a small number of ICs are needed to represent any stimulus [14]–[16]. Second, ICs are statistically independent of each other, allowing easy handling of PDs of natural scenes [30]. Third, the filters of the ICs of natural scenes are very much like the receptive fields of simple cells in V1, covering the parameter space of position, size, orientations, and spatial frequency [16], [17]. Finally, ICs of natural chromatic images, stereoscopic images, and movies have revealed many aspects of early visual processing [36]–[39].To model the context-mediated PDs in static natural scenes, we used a center-surround configuration in which the scene patch within the circular center serves as the target and the scene patch in the annular surround as the context. We sampled a large number of scene patches using this configuration from the Netherland grey image database [17] and McGill calibrated color image database [40] of natural scenes. Thus, each sample is a pair of a patch in center (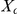) and a patch in the surrounding area (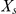) (Fig. 2A and Fig. 3A). We developed a model of natural scenes in a center-surround configure (Eq. (1)). In Eq. (1), 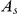, 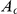, and 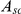 are ICs. This model allows us to calculate the ICs for the context (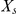) first and the other ICs of natural scenes in a center-surround configuration. It will be become clear that this model will lead to an explicit formula for the context-mediated PDs in natural scenes, i.e., the conditional PDs, 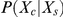.

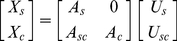
(1)ICA filters (i.e., 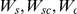) can be obtained as follows:
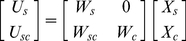
(2)


We then calculated the ICs for 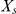 and 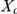 according to Eq. (1). For this purpose, we modified the FastICA algorithm [41] to achieve statistical independence within and between the components of 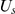 and 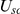. Therefore, we obtained three sets of ICs. First, the columns of 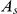 are the ICs for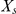. Second, the columns of 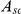 are the ICs for 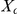 that are paired with the ICs for 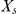. Finally, the columns of 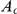 are the ICs for 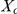 that are not paired with any ICs for 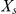.

Fig. 2B shows the paired ICs for 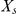 and 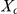 (i.e., the columns of 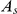 and 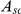) for grey images of natural scenes. The ICs for 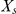 are oriented bars. The paired ICs for 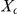 are extensions of the ICs in the surround into the circular center. For example, the paired ICs in the seventh row and the ninth column form a vertical bar across the center. The paired ICs for 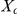 and 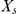 can be fitted to Gabor functions which cover the parameter space of orientation, position, size, and spatial frequency. Fig. 2C shows the ICs for 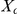 (i.e., the columns of 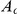) that are not paired with any ICs for 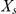. These ICs are also Gabor functions covering the parameter space of orientation, position, size, and spatial frequency. For comparison, we also obtained the ICs for 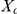 alone (Fig. 2D). Most of the ICs shown in Fig. 2C are similar to 2D, but there are some exceptions. For example, the ICs indicated by stars in Fig. 2C do not appear in Fig. 2D.

For color images of natural scenes, we applied the same procedure to the McGill calibrated color image database of natural scenes [40] to obtain three sets of ICs. Each of these three sets has chromatic and achromatic ICs. Fig. 3B shows paired chromatic ICs for 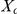 and 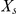. Fig. 3C shows paired achromatic ICs for 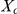 and 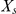. The chromatic ICs for the surround have red-green (L–M) or blue-yellow [S-(LM)] opponency. The chromatic paired ICs for the center are extensions of the ICs for the surround. The achromatic ICs are Gabor functions covering the parameter space of orientation, position, size, and spatial frequency. These results are similar to the findings obtained before [37]–[39]. Fig. 3D shows the ICs for 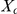, including chromatic and achromatic ICs, that are not paired with any ICs for 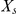. These ICs contain three channels, red/green, blue/yellow, and bright/dark. For comparison, we also obtained the ICs for center alone (Fig. 3E). Most of these ICs are similar to those shown in Fig. 3D. There are, however, some exceptions, for example, the green and yellow ICs in Fig. 3E do not appear in the Fig. 3D.

**Figure 4:**
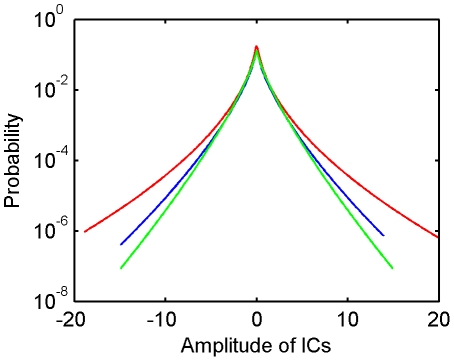
Probability distributions of three selected unpaired ICs.

The context-mediated PDs of natural scenes, i.e., the conditional PDs, 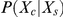, can be derived using the Bayesian formula as follows 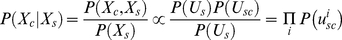
(3)where 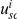 is the amplitude of the i^th^ unpaired IC for 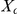. Therefore, the context-mediated PDs depend only on the unpaired ICs for 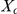, a result that is predicted by the model of natural scenes in a center-surround configuration (Eq. (1)) and will greatly simplify the computing of visual saliency of natural scenes. We modeled 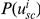 as generalized Gaussian PDs. As shown in Fig. 4, there are high peaks near zero and long tails in these PDs, indicating that only a small number of ICs are needed to encode any natural stimulus [14]–[16].

**Figure 5:**
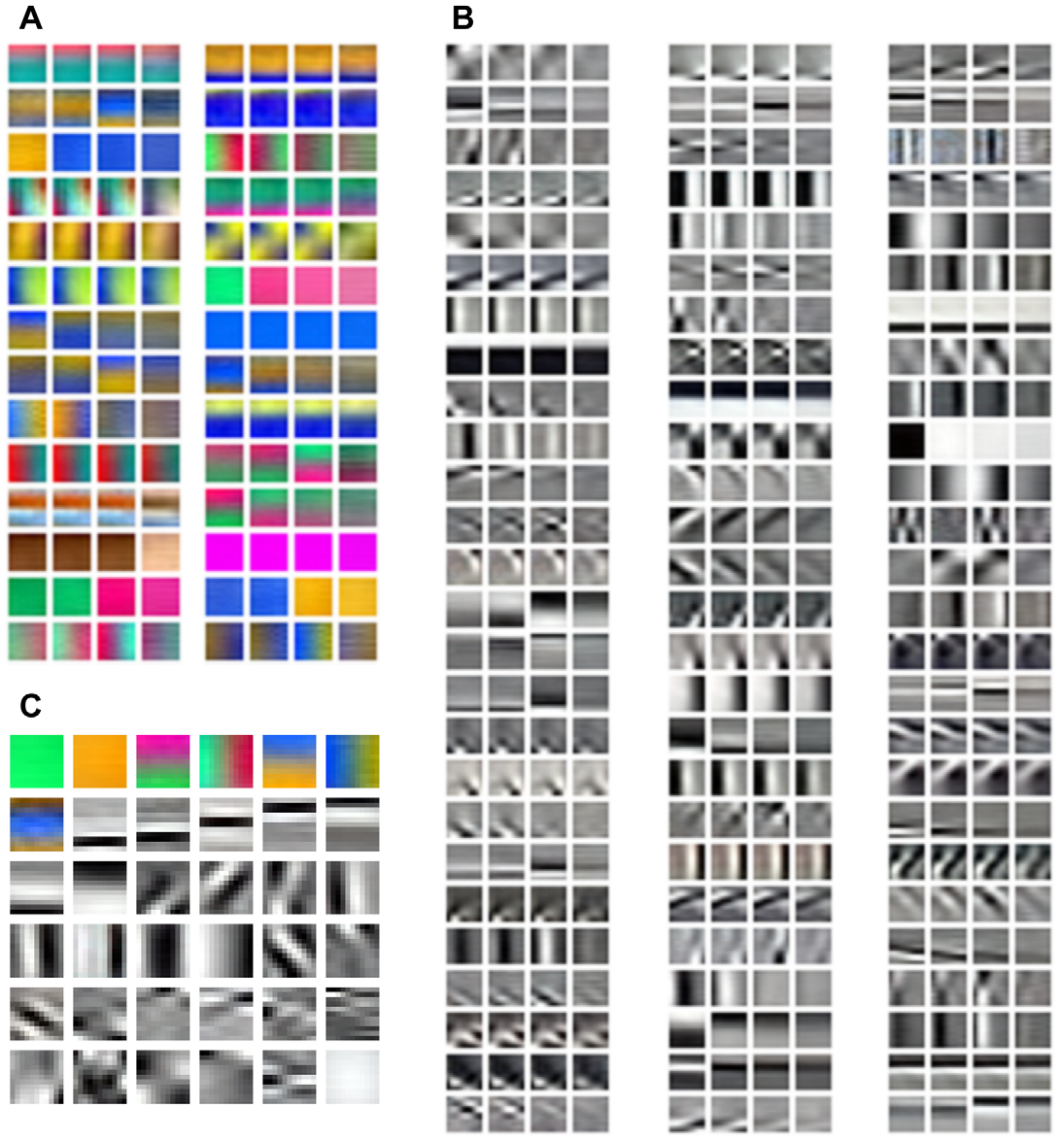
ICs of natural moving scenes. Selected paired context ICs (the left three columns of each panel) and center ICs (the right column of each panel) of 11×11×4 color patches sampled from a video database. These ICs are divided into separate red/green, blue/yellow, and bright/dark channels. (**A**) Selected 28 red/green or blue/yellow ICs. (**B**) Selected 78 bright/dark ICs. (**C**) Examples of unpaired center ICs.

To derive the context-mediated PDs in dynamic natural scenes, we used sequences of image patches in which the current frame severed as the target and the preceding frames as the context. We sampled a large number of sequences of image patches (∼490,000) from Itti's video database [34] and performed the ICA according to Eq. (1). To our knowledge, this is the first work that obtained the ICs of chromatic moving natural scenes. These ICs have three separate channels, red/green, blue/yellow, and bright/dark. Fig. 5A shows the paired chromatic spatiotemporal ICs. Fig. 5B shows the paired achromatic spatiotemporal ICs, which are consistent with the results obtained elsewhere [36]. These ICs in Fig. 5A and Fig. 5B are similar to the spatial temporal receptive fields of simple cells in primary visual cortex, which are selective for the direction and velocity of movement [42], [43]. Fig. 5C shows the unpaired ICs for the current frame, which are oriented bars and have red-green or blue-yellow opponency.

Thus, we have developed a model of the context-mediated PDs in natural scenes. This model applies equally to stereoscopic and 3D natural scenes and we can obtain the context-mediated PDs of a full range of visual variables in natural scenes. These PDs represent the most fundamental statistics of natural scenes (i.e., the statistics of natural variations of visual features and the statistics of co-occurrences of natural contexts) that any visual animal needs to deal with. If, as proposed here, these PDs have been instantiated into the visual circuitry by successful behavior in the world over evolutionary and developmental time, these PDs naturally give rise to a measure of visual saliency: 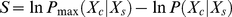
(4)


**Figure 6:**
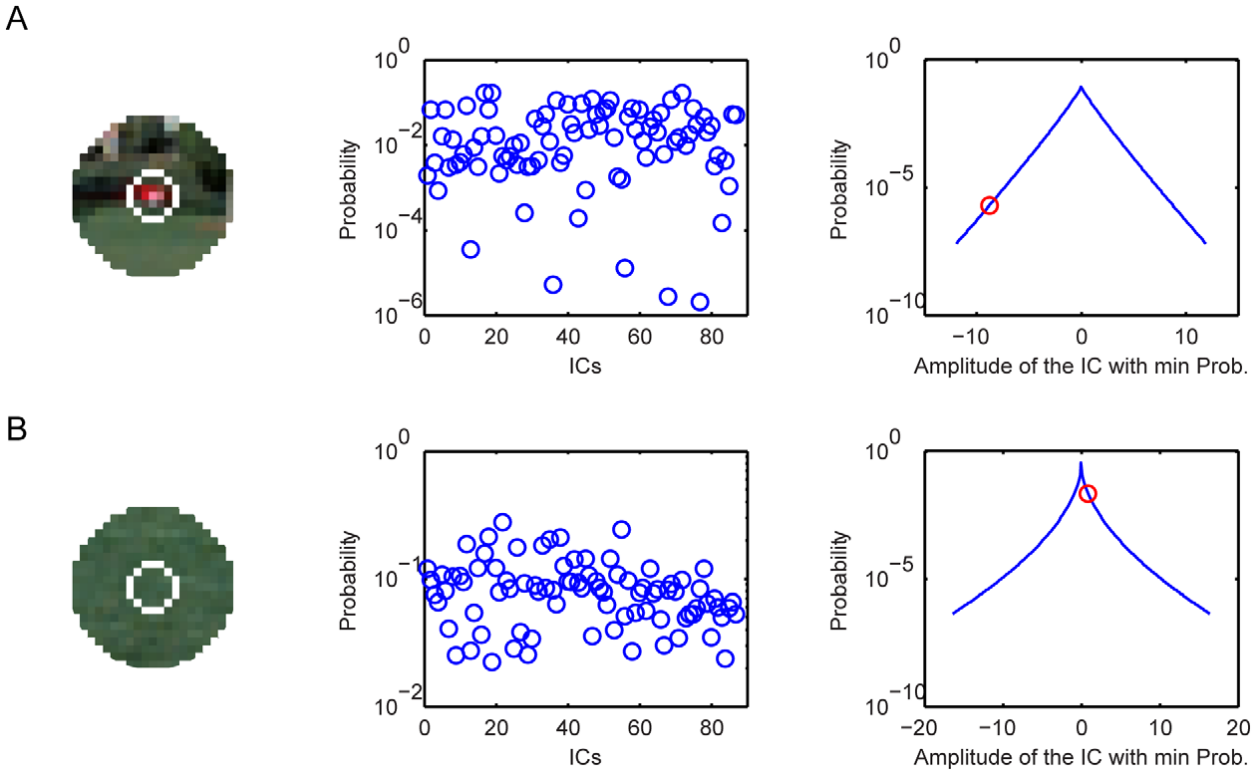
Visual saliency based on the context-mediated PDs in natural scenes. (**A**) An image patch with an salient feature at the center (left), the probabilities of all ICs (middle), and the PD of the IC that has the smallest probability (right). The red circle is the probability of the central feature. (**B**) An image patch with an non-salient feature at the center (left), and the probabilities of all ICs (middle) and the PD of the IC that has the smallest probability (right). The red circle is the probability of the central feature.

Substituting Eq. (3) into Eq. (4), we have
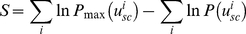
(5)where 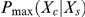
is the maximum probability of a target,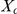, that co-occurs with a context, 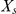, in natural scenes. Thus, if the probability of the occurrence of a target is low relative to that of the most likely occurrence in the context in natural scenes, the target is salient within the context. This fact is made clear in Fig. 6A and 6B. For a salient target in Fig. 6A, the probability of the target within the context is relatively low, and the saliency measure will be high. For a non-salient target in Fig. 6B, the probability of the target within the context is relatively high, and the saliency measure will be low.

Our model of visual saliency differs from all other models in two major ways. First, this saliency measure is based on the context-mediated PDs of a full range of visual variables in natural scenes. Most of other models are based on complex image-based feature extraction and computing [23], [24], and the context-mediated PDs in natural scenes are not used for a few models that are based on PDs in natural scenes [31]. Second, since the context-mediated PDs are related to all possible stimuli in natural scenes experienced by the visual animal over evolutionary and developmental time rather than in the current stimulus the subject is viewing, visual saliency derived here does not involve any of the image-based processing as many other models [23]–[25], [30], [32]. Next, we test whether this model of visual saliency predicts human gaze in free-viewing static and dynamic natural scenes.

### Visual saliency and human gaze in free-viewing static natural scenes

**Figure 7:**
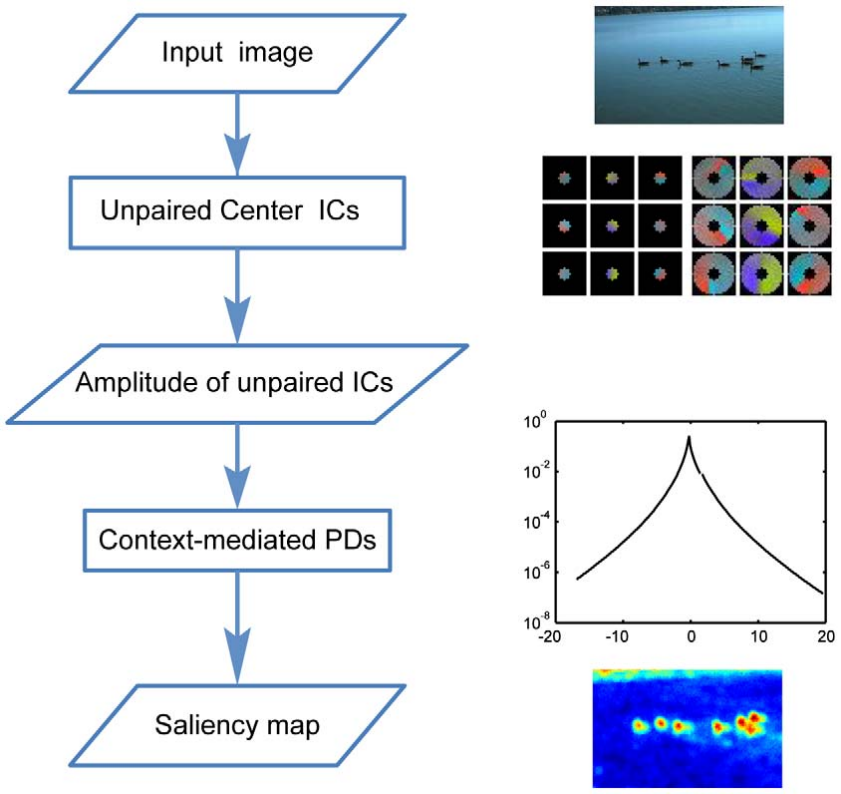
Computing visual saliency in natural scenes. Panels illustrate the steps for computing saliency at each location in any input scene. The unpaired center ICs and the context-mediated PDs are computed beforehand from a set of natural scenes. The first step is to compute the amplitudes of the unpaired ICs for the target at each location in an input scene. The second step is to compute the saliency measure based on the context-mediated PDs in natural scenes.

**Figure 8:**
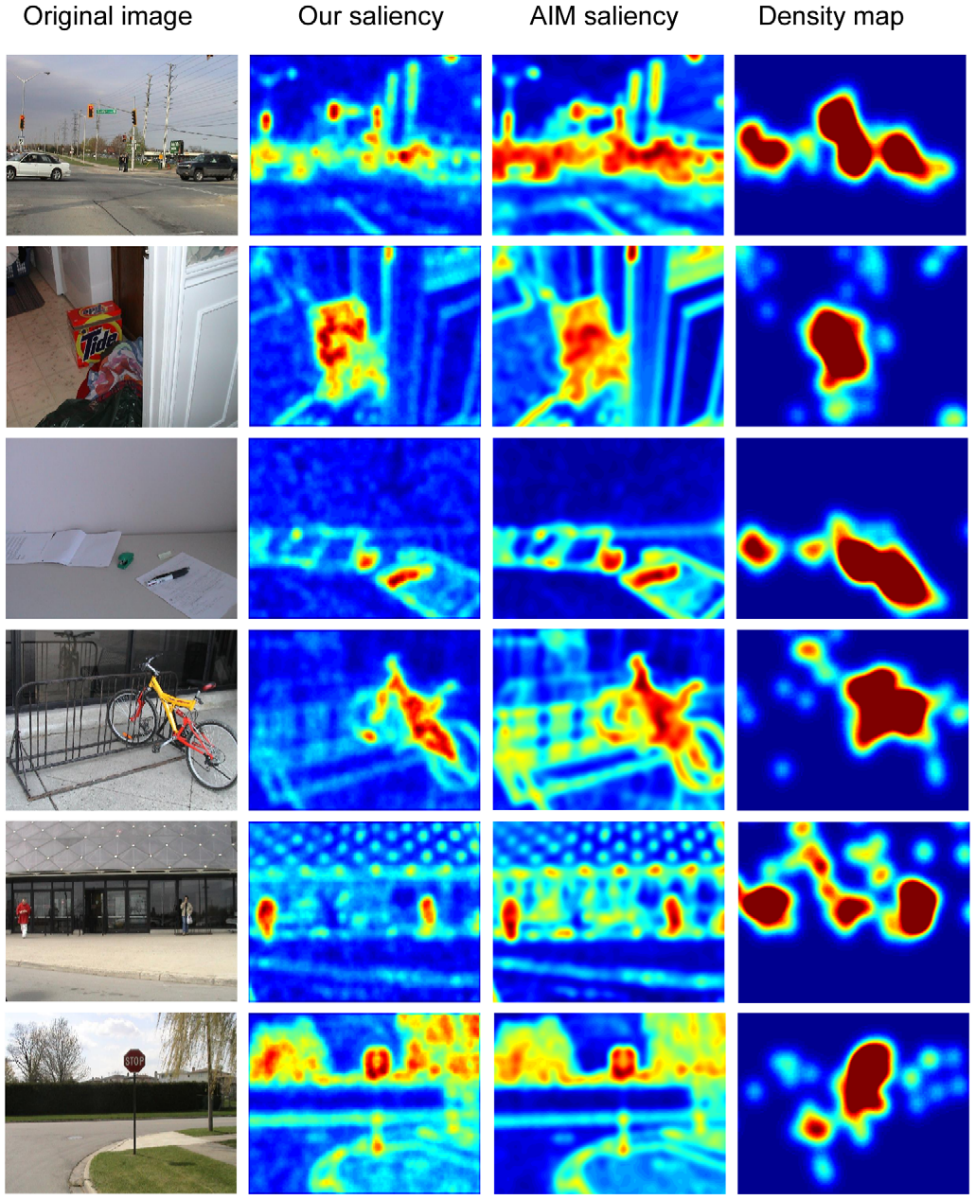
Examples of saliency maps of natural scenes. First column: input scenes. Second column, saliency maps produced by our model. Third column: saliency maps given by the AIM model. Fourth column: density maps of human fixation. Saliency is coded in color-scale (red–high saliency, blue–low saliency). According to the saliency maps, the traffic lights and the cars on the road in the first scene, the red detergent box in the second scene, the pen and the stapler in the third scene, the bicycle in the fourth scene, the two men in front of the building in the fifth scene, and the stop sign in the sixth scene appear salient.

Human gaze in free-viewing natural scenes is probably driven by visual saliency in natural scenes. To test this hypothesis, we used the procedure shown in Fig. 7 to compute saliency maps of a set of natural scenes and compared the predictions based on the saliency maps to human gaze in free-viewing these scenes. To obtain the saliency map for any scene, we computed the amplitudes of unpaired ICs for the center (i.e., 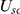) according to Eq. (2) and then the saliency measure at each location according to Eq. (5). Note that no other computation is needed to compute saliency maps in natural scenes. To compare the predictions based on saliency maps to human performance, we used the dataset of human gaze in free-viewing static natural scenes collected from 20 human subjects in free-viewing 120 images by Bruce and Tsotsos [30]. Fig. 8 shows the saliency maps based on the context-mediated PDs in natural scenes and the density maps of human gaze for six scenes. The saliency maps of Attention based on Information Maximization (AIM) model in [30] were also shown in Fig. 8. Evidently, the salient features and objects in these scenes predicted by the saliency maps accord with human observations and the saliency maps predicted by our model qualitatively matched the density maps of human gaze.

To quantitatively access how well our model of visual saliency predicts human performance, we used the receiver operating characteristic (ROC) and the Kullback–Leibler (KL) divergence measure. The ROC metric measures the area under the ROC curve. To calculate this measure, we used the saliency map as a binary classifier on every location in an input scene. We classified the locations with saliency measures greater than a threshold as fixations and the rest of the locations in the scene as nonfixated locations. By varying the threshold, we obtained an ROC curve and calculated the area under the curve which indicates how well the saliency maps predict human gaze. The KL divergence between the histogram of visual saliency sampled at fixations and the histogram of visual saliency sampled at random locations is another measure for evaluating models of visual saliency. If a model of visual saliency predicts human gaze significantly better than chance, the saliency measure computed at human fixations should be higher than that computed at random locations, leading to a high KL divergence between the two histograms.

**Figure 9:**
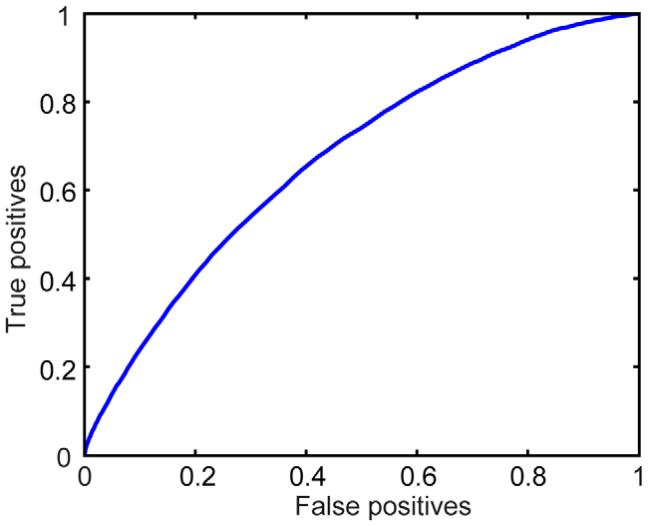
ROC curve of our saliency model.

To avoid a central tendency in human gaze [31], we used the ROC measure described in [44]. Rather than comparing the saliency measures at attended locations in the current scene to the saliency measures at unattended locations in the same scene, we compared the saliency measures at the attended locations to the saliency measures in that scene at the locations that are attended in different scenes in the dataset, called shuffled fixations. The ROC curve obtained in this way is shown in Fig. 9. The average area under the ROC curve is 0.6803, which means the saliency measures at fixations are significantly higher than the saliency measures at shuffled fixations.

**Figure 10:**
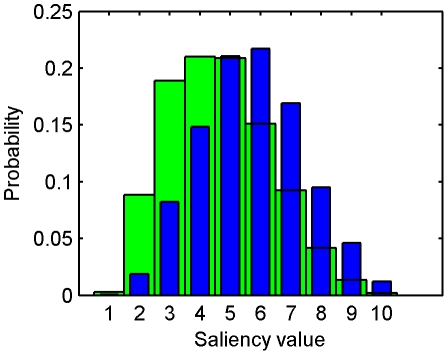
Histograms of saliency measures at the random locations (green) and fixated locations in static natural scenes (blue).

Similarly, we measured the KL divergence between two histograms of saliency measures: the histogram of saliency measures at the fixated locations in a test scene and the histogram of saliency measures at the same locations in a different scene randomly selected from the dataset [31]. The two histograms are shown in Fig. 10. The histogram of visual saliency at the fixated locations shifts to the right and thus humans tend to fixate on visual features and objects that appear salient according to our model.

Our model of visual saliency is a good predictor of human gaze during the free-viewing of static natural scenes, outperforming all other models that we tested. As shown in Table 1, our model has an average KL divergence of 0.3016 and its average ROC measure is 0.6803. The average KL divergence and ROC measure for the AIM model in [30] are 0.2879 and 0.6799 respectively, which were calculated using the code provided by the authors. The results for other models in Table 1 were given in [31]. For example, the average KL divergence and ROC measure for SUN model (ICA) are 0.2097 and 0.6682 respectively [31]. These results are surprising in two aspects. First, our model has a very simple basis (context-mediated PDs), yet it outperforms other models that are based on complex image-based feature extraction and computing [23]. Second, our model does not leverage the global statistics of a given scene, yet it outperforms other models that do [30]. Next, we examine the model's performance for moving scenes.

**Table 1:** ROC metric and KL-divergence for saliency maps of static natural scenes.

model	KL (SE)	ROC (SE)
Bruce et al (2009)[30]	0.2879(0.0048)	0.6799(0.0024)
Itti et al (1998)[23]	0.1130(0.0011)	0.6146(0.0008)
Bruce et al (2006)[49]	0.2029(0.0017)	0.6727(0.0008)
Gao et al (2007)[50]	0.1535(0.0016)	0.6395(0.0007)
Zhang: DOG (2008)[31]	0.1723(0.0012)	0.6570(0.0007)
Zhang: ICA (2008)[31]	0.2097(0.0016)	0.6682(0.0008)
Our model	0.3016(0.0051)	0.6803(0.0027)

### Visual saliency and human gaze in free-viewing natural movies

**Figure 11:**
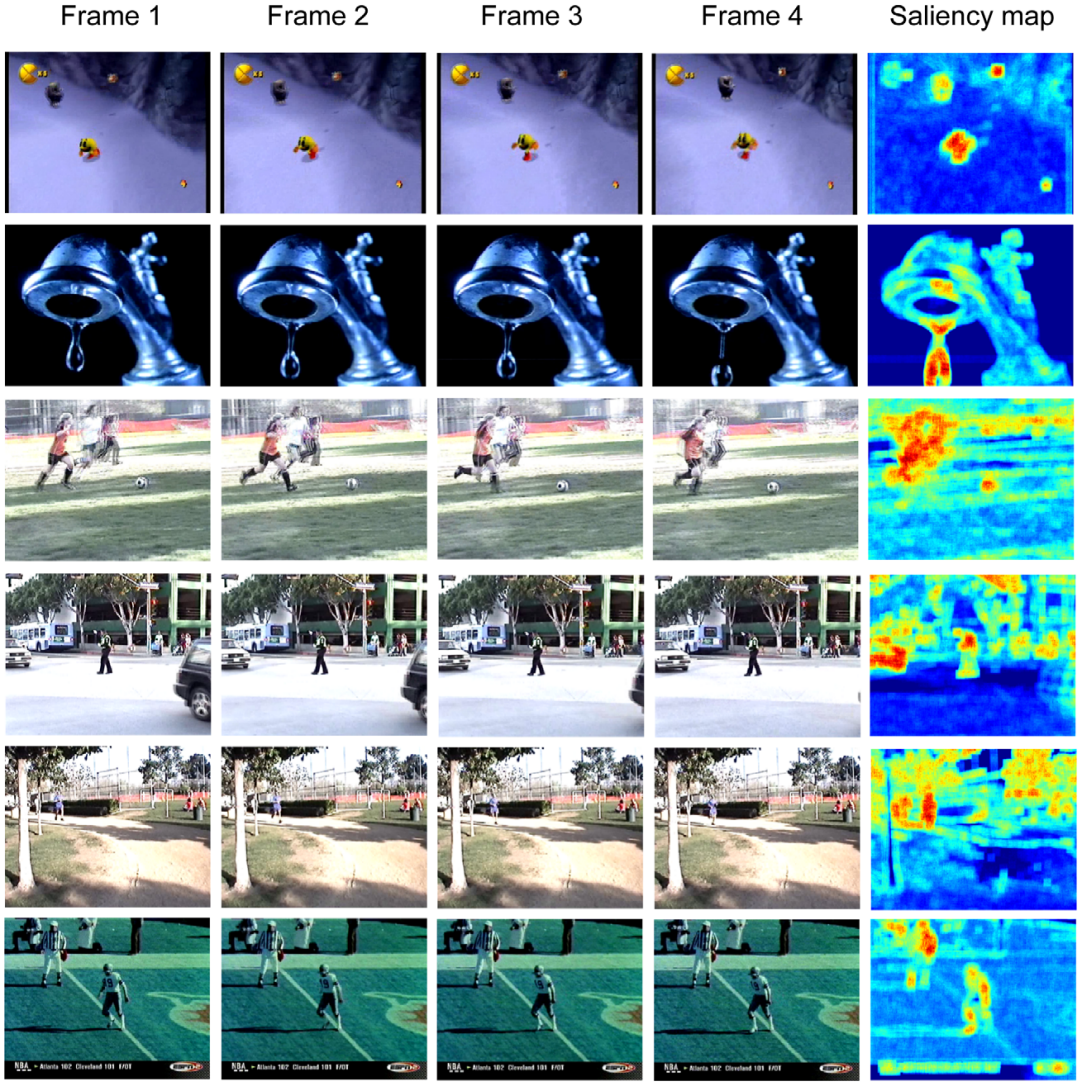
Saliency maps of dynamic natural scenes. Examples of contextual frames (the 3 left columns) and target frame (the 4th column) frames in 6 video clips and saliency maps (rightmost column). The character in the first game video, the falling water drop in the second clip, the soccer player and the ball in the third clip, the moving car and the walking policeman in the fourth clip, and the jogger in the fifth clip and the football player in the sixth clip appear salient.

We used Itti's database of human gaze in free-viewing videos[34]. The dataset contains human gaze data collected from eight human subjects in free-viewing 50 videos that included indoor scenes, outdoor scenes, television clips, and video games. We calculated visual saliency at each location in the video clips using the context-mediated PDs obtained from natural moving scenes. Fig. 11 shows the saliency maps we obtained for selected frames in 6 videos. The 3 contextual video frames and the target frame are shown to the left and the saliency maps to the right. As predicted by the saliency maps, the moving objects in these videos appear to be salient (e.g., the character in the game video, the falling water drop, the soccer player and the ball, the moving car and the walking policeman, and the jogger and the football player). These predictions accord well with human observations.

**Figure 12:**
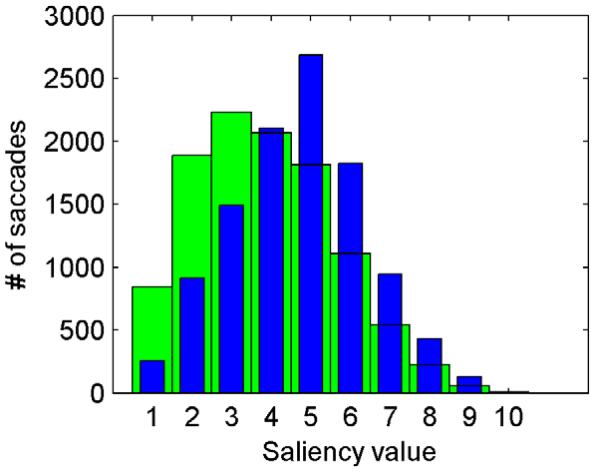
Histograms of saliency measures at the random locations (green) and fixated locations in dynamic natural scenes (blue).

Our model is a good predictor of human gaze in natural moving scenes. We calculated the KL-divergence between the histogram of saliency measures at the fixated locations in a test image and the histogram of saliency measures at the same locations in a different scene randomly selected from the dataset. As shown in Fig. 12, humans tend to gaze at visual features that have high saliency, as shown by the KL divergence measures in Table 2. The KL-divergence measure for our model is 0.3153, which is higher than the saliency metric (0.205) [23] and the surprise metric 0.241 [34], but slightly lower than the AIM model [30] (0.328). This difference may not be significant since moving natural scenes are enormously complex and a much larger dataset of human gaze is needed for evaluating models of visual saliency. The PDs in AIM model are calculated from the current video frames for which the visual saliency is computed. Therefore, for each frame, the needed PDs are recalculated, which is very time consuming. In our model, the PDs are calculated from natural scenes in advance and no other processing on the current video frames is performed.

**Table 2:** KL-divergence for saliency maps of dynamic natural scenes.

model	KL (SE)
Bruce et al (2009)[30]	0.328(0.009)
Itti et al (2009)[34]	0.241(0.006)
Zhang et al (2009)[51]	0.181
Itti et al (1998)[23]	0.205(0.006)
Our model	0.315(0.003)

## Discussion

### Contributions of this paper

First, we developed a model of the context-mediated PDs of a full range of visual variables in natural scenes. These PDs represent the most fundamental statistics of natural scenes (i.e., the statistics of natural variations of visual features and the statistics of co-occurrences of natural contexts). In this model, the context-mediated PDs in natural scenes depend only on the ICs for the target visual features that are not paired with the ICs for the contextual visual features. Using this model, we examined the context-mediated PDs of a range of visual variables in natural scenes. Second, we proposed a measure of visual saliency based on the context-mediated PDs in natural scenes. This measure of visual saliency depends on an ensemble of natural scenes that approximate the statistics experienced by humans during evolution and development. Thus, neither image-based processing (e.g., filtering, feature extraction, and normalization) nor image-based statistics (e.g., histograms of features and mutual information) is needed in this model. Finally, we conducted an extensive evaluation of our model using several datasets and found that our model is a good predictor of human gaze in free-viewing natural scenes. This is especially noteworthy since our model uses far less computational power compared to the other models we considered.

### Distinctions from other models of visual saliency

Our model of visual saliency is different from all other models. There are four classes of models of visual saliency. The first class of models do not use PDs but involve complex image-based computing that includes feature extraction, feature pooling, and normalization [23], [24]. The second class of models make use of PDs computed from the scene the subject is seeing [30]. The third class of models are based on PDs in natural scenes that are not dependent on specific contexts [31]. Finally, there is a biologically inspired neural network model [25], [26]. Our model is unique in that: 1) the PDs are not computed from any scene the subject is viewing but from an ensemble of natural scenes that presumably approximate the statistics human experienced during evolution and development, and 2) the PDs are dependent on specific contexts in natural scenes. As a result, no image-based processing is needed in our model and the computing of visual saliency is very simple.

### Neurons as estimators of the context-mediated PDs in natural scenes

These results support the notion that neurons in the early visual cortex may act as estimators of the context-mediated PDs in natural scenes. Since humans and other visual animals must respond successfully to visual stimuli whose generative sources cannot be determined in any direct way due to the inverse optics problem, the visual system can only generate perception according to the PDs of visual variables underlying the stimuli. The information pertinent to the generation of these PDs, namely, the statistics of natural visual environments, must have been incorporated into visual circuitry by successful behavior in the world over evolutionary and developmental time. Thus, an occurrence of any visual feature, is not a feature per se, but rather a sample from the PD of that visual feature in specific context in natural scenes. The goal of visual encoding is then to encode the context-mediated PDs in natural scenes. This way, any single neuron relates an occurrence of any visual variable to the underlying PD in natural scenes. These PDs are related to all possible stimuli in natural scenes experienced by the visual animals over evolutionary and developmental time.

This hypothesis is conceptually distinct from the conventional view of neurons as feature detectors, the efficient coding hypothesis [10], [11], predictive coding [45], the proposal that neurons encode logarithmic likelihood functions [46], and several recent V1 neuronal models that involve complex spatial-tempo structures [43], [47] but they don't act as estimators of PDs in natural scenes. Since the response of any single neuron encode and decode the PD of the visual variable in natural scenes, this concept is also different from probabilistic population codes [48] where populations of neurons automatically encode PDs due to a variety of noises while single neurons can have nothing to do with the PDs.

### A saliency map in the brain?

An ongoing debate in current studies on visual saliency is whether or not there should be a saliency map in the brain. Several researchers argued that there is a saliency map in the brain [23], [27]. Zhaoping argued that there is no need to have a separate saliency map since saliency can be calculated from neuronal activities within a small population [25], [26]. Other models, due to the complex computation involved, effectively assert that there is a saliency map in the brain [30]–[35]. In our model, computational units in the visual system encode the context-mediated PDs in natural scenes and thus convey saliency information explicitly. Therefore, no further complicated operations are needed to calculate visual saliency and there is no need to have a separate saliency map in the brain. To test this prediction, one can record activities of neurons in the early visual cortex in response to natural scenes and examine what additional computations are needed to derive saliency maps from the recorded neuronal responses.

### Future directions

It would be very useful to further examine whether this model of visual saliency can be applied to 3D natural scenes and to include dynamic adaptation. It would be also very useful to collect a large dataset of human gaze in free-viewing and searching dynamic, 3D natural scenes to evaluate models of visual saliency and search.

## Materials and Methods

### Natural scene statistics

To model the context-mediated PDs in natural scenes, we used the Netherland database of calibrated images of natural scenes [17] and the McGill calibrated color image database [40]. The Netherland database contains 4212 images of natural scenes obtained with a Kodak DCS420 digital camera (with a 28 mm camera lens). The images were taken in various environments (woods, open landscapes, and urban areas). The images have a resolution of 1536×1024 pixels with a pixel size of 1 minute of arc. For our purpose, we removed 344 city scenes. To reduce the computational cost, we used block averaging to reduce the image resolution to 768x512. Finally, we converted the linear scale of the luminance to the logarithmic scale, as did by several authors [17]. We sampled ∼137,000 center-surround patches from the database for ICA. The diameters of the center and the surround in Fig. 2 were 15 and 45 pixels respectively. We reduced the dimensionality of the center from 149 to 50 and the dimensionality of the context from 1368 to 200 by selecting the most significant principal components during ICA.

The McGill calibrated color image database contains 1,122 images from nine scene categories, which are flowers, animals, fruits, foliages, textures, landscapes, shadows, man-made scenes, and snow scenes. The images were taken with two Nikon Coolpix 5700 digital cameras. The images have a resolution of 786×576 pixels with each pixel having three channels (red, green, and blue). We sampled ∼110,000 center-surround patches from the images for ICA. The diameters of the center and the surround in Fig. 3 were 17 and 51 pixels respectively. We reduced the dimensionality of the center from 723 to 50 and the dimensionality of the context from 5556 to 200 by selecting the most significant principal components during ICA.

### Natural video statistics

To model the context-mediated PDs in moving natural scenes, we used the video database collected by Itti and Baldi [34]. The dataset includes 46,489 video frames in 50 video clips, each of which lasts 5.5–93.9 s and had 164 to 2814 video frames sampled at a rate of 60.27 frames per second. These video clips (with a spatial resolution of 640×480 pixels) included outdoors daytime and nighttime scenes of crowded environments, video games, and television broadcasts including news, sports, and commercials. We sampled ∼490,000 spatiotemporal volumes of size of 11×11×4 from the videos at a rate of 30.13 frames per second.

### Independent component analysis

We modified the FastICA algorithm developed by Hyvärinen [41] to perform the ICA in Eq. (1). This algorithm implements ICA by finding filters that produce extrema of the kurtosis [17]. For static color natural scenes, we whitened the input data (∼137,000 image patches) before running ICA but did not perform dimensionality reduction. The diameters of the center and the surround of the image patches were 7 and 23 pixels respectively, and the dimensionalities of the center and the surround were 87 and 1044 respectively. For natural moving scenes, before running ICA, we whitened the input data (∼490,000) and reduced the dimensionality of the center from 11×11×3 = 363 to 50 and the dimensionality of the context from 11×11×3×3 = 1089 to 200 by selecting the most significant principal components.

### Human gaze data in free-viewing static natural scenes

We used the gaze data in free-viewing static color natural scenes collected by Bruce and Tsotsos [30] to evaluate our model of visual saliency. This dataset contains human gaze collected from 20 participants in free-viewing 120 color images of indoor and outdoor natural scenes. In this free-viewing experiment, participants were instructed to free-view images of natural scenes presented on a 21-inch CRT monitor at a viewing distance of 0.75 m while their eye movements were recorded by an eye tracking apparatus.

### Human gaze data in free-viewing moving natural scenes

We used the gaze data in free-viewing moving natural scenes collected by Itti & Baldi [34]. The data were collected from 8 subjects aged 23–32 with normal or corrected-to-normal vision. Each subject watched a subset of 50 video clips and the traces of eye movement from four distinct subjects were obtained for each clip. Subjects were instructed to follow the main actors and actions in the clips and thus their gaze shifts reflected an active search for nonspecific information of subjective interest. We used two hundred calibrated traces of eye movement with a total of 10,192 saccades.
